# Feline Leukemia Virus in Cats: A Novel Rapid ELISA Assay for p27 Antigen Detection

**DOI:** 10.1155/vmi/9914340

**Published:** 2025-05-15

**Authors:** Irene Ferrero, Paolo Poletti, Enrica Giachino, Joel Filipe, Paola Dall'Ara

**Affiliations:** ^1^Department of Research and Development, Agrolabo Spa, Scarmagno, Italy; ^2^Department of Veterinary Medicine and Animal Sciences, University of Milan, Lodi, Italy

## Abstract

Feline leukemia virus (FeLV) is an oncogenic immunosuppressive virus belonging to the *Retroviridae* family and one of the most common causes of a major infectious disorder in cats that can lead to potentially fatal associated diseases with a worse prognosis. FeLV infects and replicates in hematopoietic and lymphatic cells causing anemia, lymphoma, and leukemia. Diagnosis is usually performed by clinicians using the enzyme-linked immunosorbent assay (ELISA) or lateral flow tests that detect the p27 FeLV antigen. The aim of this work was the development of the FeLVCHECK Ag ELISA, a new rapid direct sandwich ELISA assay that detects the p27 antigen of FeLV. Assay cut-off was estimated by multiple approaches, including the Youden index and the ROC curve, to obtain the optimal test performance. The new test was validated by using 112 feline sera (42 positives and 70 negatives for FeLV) against the ViraCHEK/FeLV ELISA (Zoetis) as a reference, which agreed at 97.3%, with 97.6% sensitivity (95% confidence interval (CI): 86.0%–99.9%) and 97.1% specificity (95% CI: 89.1%–99.5%). Compared with another rapid and direct ELISA, the INgezim FeLV DAS (Gold Standard Diagnostics), the agreement was 90.2%. The new ELISA is both accurate and precise, with intra and inter-assay coefficients of variation (CV) below 10%. Accelerated and real-time stability studies set the shelf life of the kit at 18 months. This study clearly suggests that the FeLVCHECK Ag ELISA can be a valuable tool in clinical practice, as it provides very rapid and reliable results without the need for sample preparation or particular laboratory equipment since all incubations are performed at room temperature.

## 1. Introduction

Feline leukemia virus (FeLV) is an oncogenic immunosuppressive virus belonging to the *Gammaretrovirus* genus and *Retroviridae* family. FeLV infection is an important cause of mortality in cats through its ability to cause immune suppression, bone marrow disorders, and hematopoietic neoplasia [1]. FeLV is an enveloped single-stranded RNA (ssRNA) virus that infects and replicates in hematopoietic precursor cells and lymphatic tissue. The genome contains three genes flanked by long terminal repeats (LTR): the group specific antigen (*gag*) gene codes for the viral structural core proteins p15 (matrix), p10 (nucleocapsid), and p12 and p27 (capsid); the polymerase (*pol*) gene codes for p14 (protease), p80 (reverse transcriptase), and p46 (integrase); the envelope (*env*) gene codes for the envelope surface glycoprotein gp70 and the transmembrane protein p15E [2, 3]. In the early stage of infection, after binding of envelope glycoproteins to cell receptors, the viral core is internalized. Then, the ssRNA is reverse-transcribed in DNA by the viral reverse transcriptase. It enters the nucleus, and it integrates into the genome of the host cell becoming “provirus” serving as a template for the production of new virions [3].

The immune system of some infected cats suppresses viral replication within a few weeks after infection, before significant involvement of the bone marrow. These cats develop a “regressive infection”: proviral DNA is present in the host cell genome, but production and shedding of virus no longer occurs, and most cats never develop clinical signs related to FeLV infection. This kind of infection can persist for life, but virus may be reactivated with immunosuppression (during pregnancy or following treatment with immunosuppressive drugs). Moreover, FeLV may cause a “focal infection,” a condition in which the immune system keeps viral replication in certain tissues (spleen, lymph nodes, or mammary gland), but not in blood or bone marrow. Furthermore, it is occasionally possible that some cats, after exposure to a low FeLV level, eliminate the infection resulting in an “abortive infection,” so no viremia occurs and virus cannot be detected using any methods. Finally, cats with bone marrow involvement may develop “progressive infection,” and in this case, the virus may exceed the ability of the host's immune system to suppress viral replication; thus, persistent viremia and progressive FeLV-related diseases may result [1, 4].

The most common signs of FeLV infection are immune suppression, anemia, and neoplasia, of which lymphoma and leukemia are the most frequent. FeLV causes neoplasia primarily as a result of insertional mutagenesis by which the virus activates proto-oncogenes or disrupts tumor suppressor genes. To date, common integration sites for the development of FeLV-associated lymphoma have been identified in six loci (*c-myc*, *flvi-1*, *flvi-2*, *fit-1*, *pim-1*, and *flit-1*) [3, 5, 6]. Because FeLV is immunosuppressive, infected cats are susceptible to a variety of secondary and opportunistic infections [1, 7].

FeLV is unstable in the environment, and therefore, transmission requires close contact between infected and healthy cats, possible by grooming or vertically during pregnancy. Indirect contact with saliva or, to a lesser extent, feces from FeLV-infected cats, can also be sufficient to transmit the infection [3, 4].

Diagnosis of FeLV is usually performed detecting the p27 antigen by the lateral flow immunoassay or the enzyme-linked immunosorbent assay (ELISA) [4]. Virus isolation and the immunofluorescent assay (IFA) are principally used as confirmatory tests for viremia [8]. Since the complex logistics, FeLV isolation in cell culture is no longer used routinely, and it is therefore considered as the ultimate diagnostic criterion. The IFA is usually based on the observation of granulocytes, lymphocytes, and platelets in viremic cats. However, if a viremic cat is leukopenic or if only few peripheral leukocytes are infected, a FeLV infection may be overlooked using the IFA. Moreover, proviral DNA or FeLV RNA may be detected by polymerase chain reaction (PCR) and by the reverse transcription PCR (RT-PCR), respectively. PCR may be useful to detect cats with regressive infection, to monitor proviral levels in cats persistently infected, or to resolve the discordant results in ELISA and IFA assays [7, 8].

One of the most common serological tests to detect the presence of the p27 antigen is the ELISA. The aim of this work was the development of a new, simple, and very rapid direct sandwich ELISA assay for FeLV infection by the detection of the p27 FeLV antigen in feline samples, without the need for sample preparation, that would give correct and easy-to-interpret results.

## 2. Materials and Methods

### 2.1. Samples Employed in the Study

Feline samples (112 sera or plasma) were taken from four veterinary clinics in northern and central Italy. We did not have information about cat's clinical conditions; thus, no sample selection was performed to contemplate the broadest possible cases. All samples used were first tested with the ViraCHEK/FeLV ELISA assay (Zoetis, NJ, USA), considered the reference test, and thus classified as positive or negative for the p27 FeLV antigen. Some of these samples were highly lipemic or hemolytic.

### 2.2. Plate Preparation for Preliminary Assays

Costar high binding microplates (Thermo Fisher Scientific, Waltham, MA, USA) were utilized in the setup of the test. For coating and blocking conditions, reference was made to standardized procedures [9, 10] and based on internal company protocols and experience. ELISA microplates were initially coated with 5 or 10 μg/mL of the anti-p27 capture antibody in carbonate buffer 10 mM, pH 8 (coating buffer 1), carbonate-bicarbonate (CB) buffer pH 9.6 (coating buffer 2), or phosphate-buffered saline (PBS) (coating buffer 3) and incubated at +2°C–8°C overnight (O/N). Successively, after washing with wash buffer, plates were blocked with two commercial blocking buffers (Surmodics, Eden Prairie, MN, USA) for 1 h at room temperature (RT). The blocking buffer 1 has pH 6.5–7.5 and contains preservatives (0.02% methylisothiazolinone and 0.02% bromonitrodioxane). Instead, the blocking buffer 2 has pH 7.0–7.4, but not conservants. The plates were then drained, left to dry, and stored at +2°C–8°C. However, in another case, plates were precoated with 1 or 5 μg/mL of Poly-L-Lysine (Sigma-Aldrich, St. Louis, MO, USA), dissolved in PBS, as described in the literature [11, 12], and incubated at +2°C–8°C O/N. Then, plates were coated with the capture antibody as previously described and stored at +2°C–8°C.

### 2.3. FeLVCHECK Ag ELISA

Plates coated first with Poly-L-Lysine (1 μg/mL) then with the capture antibody (5 μg/mL) were blocked with blocking buffer 1 and used for the ELISA assay. Two types of positive control (PCs) consist of a pool of positive sera (PC1) or recombinant protein G labeled with horseradish peroxidase (HRP) (PC2) (Abcam Limited, Cambridge, UK). The negative controls (NCs) were formed by a pool of feline negative sera (NC1) or a solution of PBS and bovine serum albumin (BSA) (NC2). Undiluted samples (50 μL/well) were distributed in each well together with 50 μL of anti-p27-HRP conjugate antibody and incubated 10 min at RT. During ELISA development, two conjugate antibody diluents were considered (Diluent 1, formed by MOPS buffer for HRP conjugates at pH 6.2–6.7; Diluent 2, pH 6.5–7.5) (Surmodics, Eden Prairie, MN, USA). After a washing step with wash buffer and addition of 100 μL/well of substrate/chromogen 3′,5,5′-Tetramethylbenzidine (TMB) (Surmodics, Eden Prairie, MN, USA) that was incubated at RT in the dark for 5 min, a colorimetric reaction was developed (positive samples were colored in blue, and negative ones were colorless). Next, the reaction was stopped by adding 100 μL of stop solution consisting of sulfuric acid 0.2 M (Agrolabo S.p.A., Scarmagno, Italy); thus, the blue reaction turned into yellow. The interpretation of results was performed instrumentally by reading the optical density (OD) values at 450 nm using an ELISA microplate reader. The test was valid if PC had an OD value above 0.600 and the NC below 0.200.

### 2.4. FeLVCHECK Ag ELISA Validation

The new ELISA was validated against the ViraCHEK/FeLV ELISA (Zoetis, NJ, USA) as the reference test by testing 112 feline sera (42 positives and 70 negatives) according to the manufacturer's instructions. In this study, a number of samples in line with that utilized in other bibliographic studies for the ELISA was used [13–16].

### 2.5. Performance Comparison With the Commercial Test

A comparative study was performed between the FeLVCHECK Ag ELISA and another widely used INgezim FeLV DAS ELISA (Gold Standard Diagnostics) according to the manufacturer's instructions.

### 2.6. Reproducibility Assay

Reproducibility and precision were examined through the coefficient of variation (CV) evaluation between tests performed in a single run (intra-assay precision) or between runs (inter assay precision). Repeatability was assessed in different days and at various times in the same day, as described in the guidelines [17]. Two operators carried out assays twice a day for 14 or 15 consecutive days to consider any changes in ambient temperature; even if in the laboratory, it was always been kept between +20°C–25°C. For each sample, analysis was repeated in duplicate for a total of 56 or 60 repetitions. In addition, the average OD, standard deviation (SD), and the percentage of CV (%CV) in intra- or inter-assays were calculated.

### 2.7. Estimation of ELISA Kit Shelf-Life

Real-time (or long-term) and accelerated stability studies were performed on a single batch of ELISA plates and reagents to maintain homogeneity. In the long-term stability analysis, the ELISA kit was stored at +2°C–8°C and in the accelerated one at +37°C. Since there are no regulations about stabilities on veterinary diagnostic kits, the Arrhenius equation, reported in the Standard Guide for Accelerated Aging of Sterile Medical Device Packages ASTM F1980-21 for medical devices [18], was employed to evaluate the decay process under accelerated conditions. In our case, we set up the accelerated stability study for 6 weeks at +37°C which is equivalent to 18 months at +2°C–8°C, and time is calculated through the use of calculators found in the web based on the Arrhenius equation [19,20]. Therefore, 1 week at +37°C corresponded to 3 months at +2°C–8°C. In both stability studies, the first test was performed at the beginning of the study at time zero (T_0_). Assays were repeated weekly for the first 6 weeks (T_1_–T_6_) for the two studies and then every 3 months up to 18 months only for real-time stability. During each analysis on each sample (PC, NC, two positives, and two negatives), the percentage of remaining activity (% RA or recovery) was computed, expressed as the ratio of the obtained OD value to that of time T_0_: the minimum limit of acceptance was set at 70% [21]. The amounts of samples tested were comparable to those used in other stability studies [14, 16].

### 2.8. Cut-Off Estimation

The ELISA cut-off was measured by comparing different methods. One of these estimates the better cutoff as the OD value that corresponds to the lowest difference between the sensitivity and specificity parameters [22], while according to another [23], the optimal cutoff may be between the maximum OD value of negative samples and the minimum OD value of positive samples. Moreover, the receiver operating characteristic (ROC) curve, which maximizes the difference between the true positives and false-positives, and the Youden's index (J) were used. The J-index is calculated from the values of sensitivity and specificity and takes values from 0 (completely ineffective test) to 1 (perfect efficiency test). The best cut-off corresponds to the J index maximum value. In addition, all diagnostic settings including the accuracy, sensitivity, specificity, positive and negative predictive values (PPV and NPV), and positive and negative likelihood ratios (LR+ and LR−) were derived. On the sensitivity, specificity, and PPV and NPV parameters, the 95% confidence interval (CI) was also calculated.

### 2.9. Statistical Analysis

Statistical analysis was carried out by the *t*-Test, with a *p* value lower than 0.05 being considered significant. Moreover, the Cohen's *d* index (ratio between the difference of OD means in two set of data and the pooled SD) was applied for measuring the strength of the relation (effect size). The extent of agreement in the paired essay was calculated by Cohen's Kappa index.

## 3. Results

### 3.1. Plate Coating, Blocking, and Conjugate Antibody Selection

Costar high binding microplates were coated with 5 or 10 μg/mL of anti-p27 capture antibody diluted in three different coating buffers (Buffer 1, Buffer 2, and Buffer 3) and blocked with blocking buffer 1. Samples were tested undiluted to maximize the concentration of the antigen to be detected. ELISA assays performed by incubating both samples/conjugate antibody (1:1000) and TMB for 5 min on plates coated with 5 μg/mL of capture antibody resulted in negative results (OD values about 0.1) on positive samples. Instead, we achieved a good result with 10 μg/mL of capture antibody only when diluted in coating buffer 1 (Table 1). However, we obtained the best result on the positive sample by using coating buffer 1, a more concentrated conjugate antibody (1:250), and by increasing both incubation times from 5 to 10 min (Table 1).

To further improve the performance of the test, microplates were coated with 10 μg/mL of capture antibody in coating buffer 1 and blocked with blocking buffer 1 or blocking buffer 2. A preliminary ELISA test was performed by testing one positive sample and only conjugate (blank) with also two different conjugate diluents (Diluent 1 and Diluent 2). The undiluted samples were added with the conjugates at different dilutions from 1:250 to 1:2000, and TMB was incubated for 10 min. Blocking buffer 1 showed better results (OD values < 0.1) on blank than blocking buffer 2; thus, no binding occurred between the conjugate and the coating components. Moreover, on the positive sample, the conjugate Diluent 1 gave higher OD values on the positive sample than Diluent 2. In all cases, absorbance values were good and above 0.3 (Table 2). For these reasons, blocking buffer 1 and Diluent 1 were selected for future assays.

### 3.2. Coating Optimization

Despite good results, to improve the signal detection on positive samples and to decrease the concentration of the capture antibody for cost reasons, we performed Poly-L-Lysine plate precoating. First of all, we evaluated the absence of nonspecific binding between Poly-L-Lysine with samples (used undiluted or diluted) or controls on plates coated only with 1 μg/mL of Poly-L-Lysine. All OD values resulted low (OD < 0.2), so samples and controls had no specific binding with coating components, even when used undiluted (Supporting Table S1, verification of nonspecific binding between samples and Poly-L-Lysine). Then, plates were precoated with two solutions of Poly-L-Lysine (1 and 5 μg/mL) and coated with 5 μg/mL of the capture antibody as described previously. An ELISA preliminary assay was performed on the full test to assess the correct identification of five positives, five negatives, and controls. First, the undiluted samples were incubated together with the conjugate antibody at 1:2000 dilution for 10 min and then TMB for 5 min. The PC and NC pools of positive and negative sera, respectively, were used. In all cases, we obtained good results since the assays correctly identified positive/negative samples and controls (OD values < 0.3 for negatives/NC and OD > 0.3 for positives/PC), without appreciable difference between the two precoating types (Table 3). Thus, 1 μg/mL of Poly-L-Lysine was selected for test development.

### 3.3. PC and NC Evaluation

To guarantee the assay validity, PCs and NCs were evaluated. Initially, we tested a pool of positive feline sera at different dilutions to achieve at least an OD value greater than 0.6. In fact, a strong PC should have an OD value greater than or equal to 1.5 [13, 24], with a minimum OD value of 0.6 for test validity, as reported in bibliography [13, 24] and in ELISA assays [25–27]. To create the PC1 formed by a pool, some feline positive sera were joined and tested in the ELISA at various dilutions (1:1000–1:14,000). We chose the dilution 1:3500 because the OD value was above 1.5 (Table 4). Instead, the PC2, represented by protein G labeled with HRP, was tested as a safer alternative to the use of sera. Since protein G is known to bind the constant portions of immunoglobulins [28], in our case, we assumed that protein G could have bound to the antibody coated to the plate, so we performed ELISA assays to check the feasibility of this assumption. Protein G-HRP resulted a good PC at all tested dilutions. However, we selected the 1:12,000 because the OD value was above and equal to 1.5 and similar to that obtained with the pool of feline positive sera in the range between dilutions (1:3500–1:7000) (Table 4). In order to verify the absence of nonspecific binding, we tested the PC consisting of protein G-HRP at different dilutions (1:3500–1:20,000) on the plate coated only with Poly-L-Lysine (1 or 5 μg/mL). In the test, we also included positive/negative samples and feline positive/negative pools of sera. We obtained good results because in all cases the absorbance values were low (OD < 0.1); thus, we concluded that protein G-HRP had no specific binding with coating components, but it works only when the capture antibody is immobilized to the plate (Supporting Table S2, nonspecific binding evaluation between controls and Poly-L-Lysine).

As for the PC, initially, we tested the NC formed by a pool of feline negative sera. As reported in bibliography, the NC should not exceed an OD value of 0.3 [13, 24]. All results gave OD values lower than 0.2, with the OD value of 1:5000 similar to that of 1:10,000 dilution and slightly lower, thus clearer, compared to 1:2500. For this reason, the 1:5000 sera dilution was selected in future assays. As comparison, we tested a solution formed by PBS and BSA as an alternative to the use of sera. We performed 60 tests, and we had good results, with an average OD value of 0.050, in the OD range of the pool negative sera diluted 1:5000–1:10,000 (Table 5).

### 3.4. Evaluation of Incubation Times

To better estimate the test development time, we changed the timing of the first incubation; thus, we incubated samples and conjugate for 5, 10, and 15 min. One PC (protein G-HRP) and one positive and negative sample were tested during this preliminary assay. We observed that in the first 5 min, the reaction did not develop as much as expected, especially for the PC (the OD value of PC should be at least higher than 1.5). Instead, after 10 and 15 min, the reaction was fully developed (Supporting Figure S1, the effect of different first incubation times). Therefore, during the ELISA assay, samples could be incubated for 10 or 15 min without any particular detectability problems. However, since there was no difference, it was decided to make the assay faster, incubate the samples, and conjugate for a shorter time (10 min).

### 3.5. ELISA Cut-Off Determination

**Figure 1:**
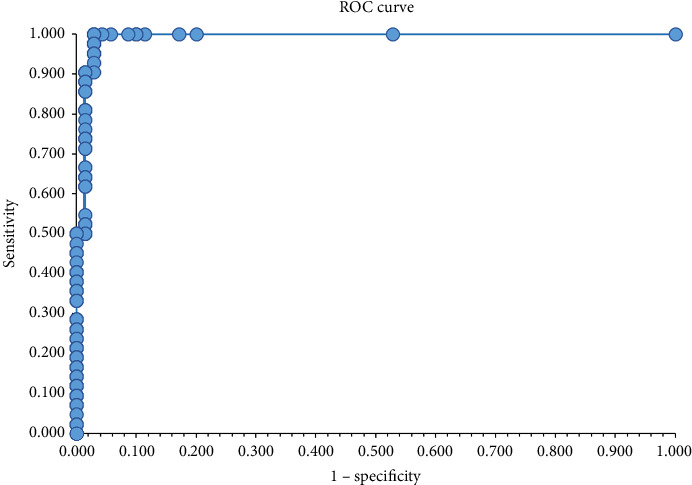
ROC curve for cut-off determination. The point closest to the upper left corner of the curve corresponds to the best cut-off with highest sensitivity (value equal to 1.0) and a low probability for a false positive result (1–specificity) of 0.029.

The cut-off was estimated by analyzing 112 samples (42 positives and 70 negatives) tested both with the FeLVCHECK Ag ELISA and the ViraCHEK/FeLV ELISA as the reference method (Supporting Table S3, FeLVCHECK Ag ELISA results used for cut-off determination and test validation). Initially, the cut-off was derived from the smallest difference between test sensitivity and specificity as reported in the literature [22] and corresponded to an OD value of 0.210 (difference minimal, equal to 0.005) with sensitivity of 97.6% and specificity of 97.1%. The same parameters were maintained until an OD value of 0.300 (Supporting Table S4, data for cut-off evaluation). In addition, the optimal cut-off may be between the maximum OD value of negative samples (OD 0.201) and the minimum OD value of positive samples (OD 0.306) [23]. Based on the ROC curve, the best cut-off corresponded to an OD value of 0.200 (Figure 1) and, according to the Youden's index, the maximum calculated J value was 0.971 that corresponded to the cut-off value with OD 0.200 (Supporting Table S4, data for cut-off evaluation). In summary, since all sensitivity and specificity parameters were retained from cut-off with OD 0.210 up to OD 0.300, an OD value of 0.300 was assumed to be the final cut-off.

### 3.6. ELISA Validation

The FeLVCHECK Ag ELISA was validated against the ViraCHEK/FeLV ELISA (Zoetis), considering the reference method, by using the same 112 samples (42 positives and 70 negatives) already tested for cut-off determination. Some of these were highly lipemic and hemolytic. We chose ViraCHEK/FeLV because it was considered the reference also in other studies involving virus isolation [29], FeLV PCR [30], and in rapid lateral-flow commercial assays [31]. The cut-off was calculated as previously described in Section 3.5. Of 112 feline samples, the FeLVCHECK Ag ELISA detected 43 positives and 69 negatives, with two false-positives (samples n. 77: OD value of 0.381; sample n. 95: OD value of 0.987) and one false-negative (n. 98: OD value of 0.201, “doubtful”) (Supporting Table S3, FeLVCHECK Ag ELISA results used for cut-off determination and test validation). Compared with ViraCHEK/FeLV, the developed ELISA agreed at 97.3%, sensitivity was 97.6% (95% CI: 86.0%–99.9%), and specificity was 97.1% (95% CI: 89.1%–99.5%). The Cohen's Kappa was 0.943, the index of very good agreement (Table 6). The PPV was 95.3%, and the NPV was 98.6%. Moreover, LR+ and LR− were 34.167 and 0.025, respectively.

### 3.7. Reproducibility Study

The PC (protein G-HRP), the NCs consisting of the pool of feline negative sera (NC1) and PBS/BSA-based solution (NC2), and two positive and two negative samples were tested in duplicate, twice a day, for 14 or 15 consecutive days (56 or 60 tests for each sample) (Supporting Table S5, reproducibility study). The FeLVCHECK Ag ELISA resulted precise and accurate, with intra- and interassay %CV lower than 20%, as reported in bibliography [21, 32] (Table 7); Supporting Table S6, reproducibility assay: intra- and interassay CV).

### 3.8. Comparative Investigation

Good agreement (90.2%, Cohen's Kappa: 0.786) resulted from the comparison between the FeLVCHECK Ag ELISA with the widely used INgezim FeLV DAS ELISA (Gold Standard Diagnostics) [33, 34]. Of 43 positive samples, the INgezim FeLV DAS ELISA identified 34 samples, while 9 resulted false-negatives. Moreover, of 69 negative samples, the INgezim FeLV DAS ELISA identified 67 true-negatives, while two were false-positives (Table 8; Supporting Table S7, results of the comparative study). Statistical analysis performed by the *t*-Test (*p* value < 0.05) on mean absorbance values between the two assays found a statistically significant difference only for positive samples. However, the effect size calculated by Cohen's *d* gave a value of 0.773 for positive samples, the index of only moderate difference (data not shown). Instead, compared to ViraCHEK/FeLV, the INgezim FeLV DAS ELISA detected 9 false-negatives and 3 false-positives; thus, the proportion of agreement was 89.3% (Cohen's Kappa: 0.765) (Table 8).

### 3.9. Stability Studies

In all tested time points, the percentage of remaining activities (% RA) were above 70% in both accelerated (Supporting Table S8, detailed data of the accelerated stability study) and real-time (Supporting Table S9, detailed data of the real-time stability study) stabilities, proving that all the reagents in the kit are stable for 6 weeks at +37°C and for 18 months at the storage temperature +2°C–8°C.

## 4. Discussion

The ELISA for the FeLV antigen represents one of the most widely used test for FeLV detection [4]. The ViraCHEK/FeLV ELISA was chosen as the reference method for our FeLVCHECK Ag ELISA since it is a qualitative assay with high sensitivity (≥ 94.9%) and high specificity (≥ 98.4%) compared with virus isolation [29] or FeLV PCR [30] and because it was chosen as the reference also for other commercial tests such as SNAP Feline Triple (IDEXX, ME, USA), WITNESS FeLV-FIV (Zoetis, NJ, USA), and VetScan Feline FeLV/FIV (Abaxis, CA, USA) [31]. Compared to ViraCHEK/FeLV, the new FeLVCHECK Ag ELISA has high sensitivity (97.6%, 95% CI: 85.9%–99.9%) and high specificity (97.1%, 95% CI: 89.1%–99.5%).

During assay development, we improved the signal on positive samples by precoating plates with Poly-L-Lysine to increase adherence of the capture antibody to the wells. It is known that Poly-L-Lysine, a synthetic amino acid chain positively charged, is widely used in histochemical applications as a coating agent to enhance cell attachment and adhesion on solid surfaces (plastic or glass) through electrostatic interaction between negatively-charged ions of the cell membrane and positively-charged surface ions of the Poly-L-Lysine [35]. From data present in bibliography, Poly-L-Lysine precoating was used in some ELISA tests [11, 12].

During initial phases of test development, ELISA kit controls were formed by pools of feline sera. However, other commercial reagents (protein G-HRP as PC and PBS/BSA-based solution as NC) were evaluated for reasons related to the future production need and safety of the ELISA kit. First, the availability, especially of positive samples, is limited, and the amount of undiluted serum/plasma to be collected and placed in the final kit would be too much, thus unsustainable at the production level. In addition, the application of positive feline samples as a control could lead to variance due to the kind of samples used (strongly or weakly positive). This factor might considerably influence the type of PC, leading to a lack of uniformity between batches. In contrast, available commercial reagents used at specific concentrations result in less variability than those obtained with samples. In fact, tests performed on protein G-HRP during quality control of FeLVCHECK Ag ELISA batches produced between the years 2023 and 2025 resulted in a maximum 2% of intra-assay %CV and in slightly more than 1% of interassay %CV. Both %CVs were much lower than 20% limit as reported in bibliography [21, 32], demonstrating the test precision by using this PC even on a commercial level. Finally, to safeguard operator safety, it is generally recommended to avoid the use of potentially infectious samples in the kit.

The PC in a test should always show a positive result and serves to indicate that the assay was performed correctly. In this context, we developed a PC formed by protein G that should allow to verify the presence and correct functionality of the coated antibody by binding to the capture antibody immobilized on the plate. Protein G, expressed by streptococcal bacteria, binds the constant portions of immunoglobulins [28]; thus, it is widely used for immunoprecipitation assays [36, 37] and antibody purification [38–40]. Protein G was already applied in some ELISA tests as conjugate to detect IgG antibodies of various host species [41–44].

After preliminary assays, we demonstrated that protein G labeled with HRP might be an excellent PC in the FeLVCHECK Ag ELISA because of the good performances, similar to that obtained with the pool of feline positive sera. In fact, in our ELISA, we have included a strong PC with an OD value above or higher than 1.5, as described in the literature [13, 24] choosing a minimum OD of 0.6 for the test validity as in other ELISA assays [25–27]. Regarding the NC, PBS-based solutions are already used as NC in ELISA tests developed both for research purposes and in commercially available assays [45–49]. In our developed ELISA assay, the NC formed by a PBS/BSA-based solution gave always results below the OD limit of 0.3 [13, 24] similar to that obtained from feline negative sera, confirming that it may be used as NC in our ELISA.

Good agreement (90.2%, Cohen's Kappa: 0.786) resulted from the comparative study between the FeLVCHECK Ag ELISA with the commercially used INgezim FeLV DAS ELISA (Gold Standard Diagnostics) [33, 34] resulting in 9 false-negatives and two false-positives. Compared to ViraCHEK/FeLV as the reference, the INgezim FeLV DAS ELISA detected 9 false-negatives and 3 false-positives, while the FeLVCHECK Ag ELISA detected one false-negative and two false-positives. One of two false positives in our ELISA resulted positive also with the INgezim FeLV DAS ELISA. Given the previously reported specificity of ViraCHECK/FeLV equal to 98.4% [31], this result may represent a ViraCHEK/FeLV false-negative. Similarly, the false-negative sample in our ELISA resulted negative also with the INgezim FeLV DAS ELISA (Gold Standard Diagnostics), thus maybe representing a false-positive in ViraCHECK/FeLV. No other verifications were performed.

One of the reasons that can explain false-positive reactions is due to nonspecific binding of samples to the solid phase. At this purpose, we demonstrated no aspecific binding with coating components between samples and controls. However, this step is sample-depended and hardly predictable. Another possible reason for a false-positive result can be the presence of antibodies against antigens that mimic the FeLV epitopes and therefore cause cross-reactivity. A study in the literature reported that the anti-p27 monoclonal antibodies reacted with the polyproteins p95 gag-fes from Gardner-Arnstein (GA) and p83 gag-fgr from Theilen-Pedersen (TP1) of feline sarcoma virus (FeSV). The anti-p27 monoclonal antibodies also reacted with murine leukemia virus (MuLV) p30 and p28 of RD114 virus but not with RSV, MMTV, or BLV viruses. These results indicate that the part of the p27 gag gene is preserved in GA and TP1 of FeSV and encodes interspecies-specific p27 determinants [50]. Moreover, in other studies, it was reported that in serological tests for the FeLV antigen, false-positive reactions may be associated with antibodies in cat serum directed against mouse immunoglobulins, detected in 0.35% of sera tested [30, 51]. In our case, the capture and the conjugate antibodies were produced in mouse, and this may explain our false-positive cases. Moreover, previous vaccinations against FeLV can lead to positive results [52]. Assays that result positive with a point-of-care (POC) test but negative with PCR may indicate a false-positive in an uninfected cat or could indicate FeLV exposure resulting in a focal or regressive infection. Some studies identified p27 antigen-positive cats without evidence of viremia or proviral DNA [53].

Regarding false-negative reactions, sample preservation is very important. In fact, if specimens are placed for too long, the immunoreactivity may be weakened, and false negatives may occur. In order to overcome the abovementioned interference, samples determined by the ELISA should be freshly collected.

In the FeLVCHECK Ag ELISA, no interaction was noticed on highly lipemic or hemolytic samples tested and collected in different types of anticoagulants. Although hemoglobin, bilirubin, or lipid in the samples was reported to not affect performance of the FeLV ELISA [54], the presence of ELISA-interfering substances, including drugs or natural compounds, in cats with profound inflammation or immune activation should be explored. In this instance, we have not carried out extensive studies to investigate cross-reactivity reactions, partly because of the lack of available representative samples, hard to collect because they should derive from animals affected by various diseases whose distribution also depends on endemic or seasonal status in different countries. In order to further improve the performance of the test, despite the difficulties in obtaining samples, the cross-reactivity study should be expanded in the future. Considering the patient's clinical symptoms and history, false-positive and false-negative FeLV tests should be retested by the ELISA and, in any case, confirmed by some other assay, such as virus isolation, immunofluorescence, or PCR.

The temperature of +37°C is usually used for accelerated stability to speed up the chemical/physical reactions of reagents and, for this reason, is the preferred method to establish the expiry date of the product rapidly, rather than waiting a long time for the degradation to happen [55, 56]. In fact, during transport or storage, many unforeseen factors may arise, such as a break in the cold chain, which may adversely affect product quality and performance. Based on national requirements, it may be necessary to conduct stability tests at defined temperatures that reflect the climatic zone of the country where the product is to be marketed. The climatic regions in which products are sold determine the storage conditions to be used. When a product is stable at storage conditions typical of the warmer climate area, it is automatically appropriate for use also in the colder one [57, 58]. The temperature of +37°C mimics that of the hotter climatic areas, and therefore, after the stability tests have been completed, the product can be released in all climatic zones, even those hot, dry, and very humid. Unlike pharmaceuticals or veterinary drugs, for which stability test guidelines are in place [59, 60], the shelf-life regarding veterinary diagnostic kits do not mention any specific legislation. Thus, in the lack of standards, we referred to existing methods used for medical devices [18]. Based on this guidance, we proved that the new kit was stable both after 6 weeks at +37°C and for 18 months at refrigerated temperature, making the product appropriate for use even under conditions of possible cold chain failure.

Since the FeLVCHECK Ag ELISA tolerates variations in sample incubation times up to 5 min longer and temperatures up to 37°C, it is a robust test. The new assay is also accurate with intra- and interassay %CV lower than 20%, as reported in the general guidelines [21, 32].

Similar to other tests developed by the same research group [61], no reports of test dysfunction have been received by the company from end-users since the product was placed on the market (2 years). The proper performance of the test is demonstrated by a recently reported study in the literature, in which feline samples were tested for the FeLV p27 antigen first with the lateral flow test and then confirmed with the FeLVCHECK Ag ELISA [62].

## 5. Conclusions

The FeLVCHECK Ag ELISA is a new assay that reveals FeLV infection by p27 antigen detection characterized by high sensitivity and specificity. It is accurate and rapid, providing reliable results very quickly (15 min) and saving time since the samples and the conjugated antibody are added together in the first incubation. The new assay does not require sample preparation or particular laboratory equipment since all assays are performed at RT. The only instrument needed is a spectrophotometer to read the results. The new ELISA contains safe reagents for the end user and has a long-term shelf life of up to 18 months if kept refrigerated. All these properties render this assay optimized for use in veterinary medicine.

## Figures and Tables

**Table 1:** Preliminary ELISA assay results.

Conjugate	Incubation times (min)	Samples	Coating buffer 1		Coating buffer 2		Coating buffer 3	
			5 μg/mL	10 μg/mL	5 μg/mL	10 μg/mL	5 μg/mL	10 μg/mL
1:1000	5/5	Positive	0.157	0.343	0.096	0.104	0.069	0.092
		Negative	0.037	0.038	0.037	0.037	0.043	0.038

1:250	10/10	Positive	0.331	1.156	0.254	0.330	0.189	0.399
		Negative	0.056	0.069	0.049	0.058	0.069	0.082

*Note:* Plates were coated with the anti-p27 capture antibody (5 or 10 μg/mL) in carbonate buffer 10 mM with pH 8 (coating buffer 1), carbonate-bicarbonate (CB) buffer with pH 9.6 (coating buffer 2), and phosphate-buffered saline (PBS) (coating buffer 3) and blocked with blocking buffer 1. One positive and one negative undiluted sample were incubated together with the anti-p27-HRP conjugate antibody (1:1000 or 1:250 dilutions in Diluent 1). Incubation times during first and second ELISA phases: 5 or 10 min at RT.

**Table 2:** Blocking buffer and conjugate antibody diluent selection.

Samples	Conjugate dilution	Diluent 1		Diluent 2	
		Blocking buffer 1	Blocking buffer 2	Blocking buffer 1	Blocking buffer 2
Positive (*n* = 1)	1:250	1.884	2.370	1.701	2.406
	1:500	1.362	1.949	1.118	1.732
	1:1000	0.888	1.410	0.722	1.054
	1:2000	0.495	0.737	0.361	0.524

Blank (*n* = 1)	1:250	0.094	0.139	0.090	0.107

*Note:* OD values were obtained on plates coated with 10 μg/mL of the capture antibody in coating buffer 1 and blocked with blocking buffer 1 or blocking buffer 2. One positive sample and one blank (only conjugate antibody) were incubated for 10 min at RT with the conjugate antibody (1:250–1:2000 dilutions) in Diluent 1 or Diluent 2. TMB substrate incubation time: 10 min.

**Table 3:** Verification of FeLVCHECK Ag ELISA functionality.

Poly-L-Lysine (μg/mL)	Positive samples					Negative samples					PC	NC
	1	2	3	4	5	1	2	3	4	5		
1	1.644	2.508	0.344	1.062	1.717	0.060	0.057	0.044	0.044	0.052	2.675	0.054
5	1.767	2.634	0.451	1.129	1.552	0.054	0.051	0.054	0.043	0.049	2.598	0.056

*Note:* ELISA was performed by testing 5 positive and 5 negative samples on plates precoated with 1 or 5 μg/mL of Poly-L-Lysine and coated with the capture antibody. Data in tables represent the OD values. PC: positive control (pool of feline positive sera); NC: negative control (pool of feline negative sera).

**Table 4:** Positive control evaluation in the FeLVCHECK Ag ELISA.

PC dilutions	PC1	PC2
1:1000	3.157 ± 0.002 (*n* = 2)	n.d.
1:3500	2.757 ± 0.002 (*n* = 2)	2.687 ± 0.086 (*n* = 4)
1:5000	n.d.	2.622 ± 0.057 (*n* = 6)
1:6000	n.d.	2.615 ± 0.018 (*n* = 2)
1:7000	1.463 ± 0.002 (*n* = 2)	2.523 ± 0.055 (*n* = 10)
1:10,000	n.d.	2.249 ± 0.088 (*n* = 10)
1:12,000	n.d.	2.026 ± 0.022 (*n* = 3)
1:14,000	0.712 ± 0.001 (*n* = 2)	1.755 ± 0.065 (*n* = 8)
1:20,000	n.d.	1.356 ± 0.129 (*n* = 3)

*Note:* Results are expressed as mean OD ± standard deviation (SD). The PCs were tested at dilutions ranging from 1:1000 to 1:20,000. PC1: positive control (pool of feline positive sera); PC2: positive control (protein G-HRP); *n*: number of tests.

Abbreviation: n.d., not determined.

**Table 5:** Negative control evaluation in the FeLVCHECK Ag ELISA.

Sample	Dilution	Mean OD ± SD
NC1	1:2500	0.063 ± 0.001 (*n* = 2)
	1:5000	0.052 ± 0.001 (*n* = 2)
	1:10,000	0.051 ± 0.001 (*n* = 2)

NC2	—	0.050 ± 0.001 (*n* = 60)

*Note:* Results are expressed as mean OD ± standard deviation (SD). NC1: pool of feline negative sera diluted 1:2500–10,000; NC2: PBS/BSA-based solution; *n*: number of tests.

**Table 6:** Validation results of the FeLVCHECK Ag ELISA (Agrolabo) against the reference ViraCHEK/FeLV ELISA (Zoetis).

FeLVCHECK Ag ELISA (Agrolabo)	ViraCHEK/FeLV ELISA (Zoetis)–reference test		
	Positive	Negative	Total
Positive	41 (a)	2 (b)	43
Negative	1 (c)	68 (d)	69
Total	42	70	112

*Note:* Total number of samples analyzed: 112. (a) true-positives; (b) false-positives; (c) false-negatives; (d) true-negatives. Proportion of agreement (a + d/a + b + c + d): 97.3%; Cohen's Kappa: 0.943; Sensitivity: 97.6%; Specificity: 97.1%.

**Table 7:** Summary of the reproducibility study.

Sample	*N*	Mean OD	SD	%CV	Intra-assay %CV		Interassay %CV	
					Minimum	Maximum	Minimum	Maximum
PC	56	3.947	0.063	1.606	0.000	2.590	0.213	1.531
NC1	56	0.055	0.005	9.195	1.150	9.801	2.034	6.524
NC2	60	0.050	0.001	1.610	0.000	4.201	0.700	2.800
Sample 1 (+)	56	1.423	0.083	5.852	0.268	9.144	0.680	6.933
Sample 2 (+)	56	0.830	0.077	9.307	0.257	8.607	0.576	4.940
Sample 3 (−)	56	0.055	0.004	7.654	0.000	8.918	1.274	7.586
Sample 4 (−)	56	0.042	0.004	8.870	0.000	10.607	1.684	9.367

*Note:* PC: positive control (protein G-HRP); NC: negative control (NC1: pool of negative sera, 1:5000; NC2: PBS/BSA-based solution); N: number of tests; %CV: percentage of coefficient of variation; *n*: number of assays.

Abbreviations: n.d., not determined; OD, optical density; SD, standard deviation.

**Table 8:** Comparison results between the FeLVCHECK Ag ELISA (Agrolabo) and the INgezim FeLV DAS ELISA (Gold Standard Diagnostics) or the ViraCHEK/FeLV ELISA (Zoetis).

INgezim FeLV DAS ELISA (GSD)	FeLVCHECK Ag ELISA (Agrolabo)			ViraCHEK/FeLV ELISA (zoetis)		
	Positive	Negative	Total	Positive	Negative	Total
Positive	34 (a)	2 (b)	36	33 (a)	3 (b)	36
Negative	9 (c)	67 (d)	76	9 (b)	67 (d)	76
Total	43	69	112	42	70	112

*Note:* Proportion of agreement of the INgezim FeLV DAS ELISA (a + d/a + b + c + d): 90.2% (FeLVCHECK Ag ELISA Agrolabo) and 89.3% (ViraCHEK/FeLV ELISA); (a) true-positives; (b) false-positives; (c) false-negatives; (d) true-negatives.

## Data Availability

Data are available in this published article and as Supporting Information. Datasets generated during the current study are also available from the corresponding author on reasonable request.

## Supporting Information

Supporting Table S1. Verification of nonspecific binding between samples and Poly-L-Lysine. Supporting Table S2. Nonspecific binding evaluation between controls and Poly-L-Lysine. Supporting Table S3: FeLVCHECK Ag ELISA results used for cut-off determination and test validation. Supporting Table S4: Data for cut-off evaluation. Supporting Table S5: Reproducibility study. Supporting Table S6: Reproducibility assay: intra- and interassay coefficient of variation. Supporting Table S7: Results of the comparative study. Supporting Table S8: Detailed data of the accelerated stability study. Supporting Table S9: Detailed data of the real-time stability study. Supporting Figure S1: Effect of different first incubation times.
