# Supplementary material for: Feline Leukemia Virus in Cats: A Novel Rapid ELISA Assay for p27 Antigen Detection
Source: Vet Med Int. 2025 May 15;2025:9914340. doi: 10.1155/vmi/9914340 (PMC12097850; doi:10.1155/vmi/9914340)
Supplement: Supporting Information — Additional supporting information can be found online in the Supporting Information section. [file 9914340.f1.pdf]

Supplementary Table S1: Verification of nonspecific binding between samples and Poly-L-Lysine. Plates were coated only with 1 μg/mL of Poly-L-Lysine. Samples (one positive and one negative) were tested undiluted and diluted (1:2 - 1:32). Conjugate: anti-p27-HRP antibody 1:2000. NC: negative control (pool of feline negative sera); PC: positive control (pool of feline positive sera). Incubation times: 10 minutes (samples/controls and conjugate), 5 minutes (TMB).

**Samples**	**Sample dilution**	**Positive sample**	**Negative sample**
Sample	Undiluted	0.043	0.044
	1:2	0.039	0.037
	1:4	0.039	0.038
	1:8	0.035	0.035
	1:16	0.039	0.039
	1:32	0.035	0.034
NC	1:5000	0.037	0.036
PC	1:3500	0.039	0.039

Supplementary Table S2: Nonspecific binding evaluation between controls and Poly-L-Lysine. Plates were coated only with 1 µg/mL and 5 µg/mL of Poly-L-Lysine. PC1: Protein-G-HRP (1:3500 – 1:20,000); PC2: pool of feline positive sera 1:3500; NC1: pool of feline negative sera 1:5000; NC2: PBS/BSA-based solution. Samples: n.1 (positive), n.2 (negative), tested undiluted.

**Sample**	**Dilution**	**Poly-L-Lysine**	
		**1 µg/mL**	**5 µg/mL**
PC1	1:3,500	0.036	0.039
	1:7,000	0.051	0.054
	1:10,000	0.046	0.044
	1:14,000	0.039	0.037
	1:20,000	0.038	0.036
PC2	1:3,500	0.044	0.040
NC1	1:5,000	0.062	0.072
NC2	-	0.043	0.055
Sample 1 (+)	-	0.038	0.036
Sample 2 (-)	-	0.051	0.049

Supplementary Table S3: FeLVCHECK Ag ELISA results used for cut-off determination and test validation. The developed ELISA (Agrolabo) was validated against the reference ViraCHEK/FeLV ELISA (Zoetis) by testing 112 feline samples. Samples with discordant results are marked with an asterisk and highlighted in bold. OD: optical density; POS: positive; NEG: negative.

Samples	ViraCHEK/FeLV (Zoetis)	FeLVCHECK Ag ELISA (Agrolabo)	
		OD values	Results
1	NEG	0.047	NEG
2	POS	2.612	POS
3	POS	0.711	POS
4	NEG	0.037	NEG
5	NEG	0.035	NEG
6	POS	1.466	POS
7	NEG	0.114	NEG
8	POS	1.310	POS
9	NEG	0.039	NEG
10	NEG	0.040	NEG
11	NEG	0.034	NEG
12	POS	1.126	POS
13	NEG	0.036	NEG
14	NEG	0.043	NEG
15	NEG	0.040	NEG
16	NEG	0.046	NEG
17	NEG	0.047	NEG
18	NEG	0.035	NEG
19	NEG	0.038	NEG
20	POS	2.606	POS
21	NEG	0.039	NEG
22	POS	1.363	POS
23	NEG	0.046	NEG
24	NEG	0.046	NEG
25	NEG	0.037	NEG
26	NEG	0.036	NEG
27	NEG	0.036	NEG
28	NEG	0.039	NEG
29	POS	0.498	POS
30	NEG	0.047	NEG
31	NEG	0.044	NEG
32	NEG	0.039	NEG
33	NEG	0.037	NEG
34	NEG	0.047	NEG
35	NEG	0.038	NEG
36	NEG	0.036	NEG
37	NEG	0.129	NEG
38	NEG	0.037	NEG
39	NEG	0.064	NEG
40	NEG	0.038	NEG
41	POS	0.779	POS
42	POS	0.820	POS
43	NEG	0.036	NEG
44	POS	1.050	POS

Continued in the next page

Supplementary Table S3: Continued.

Samples	ViraCHEK/FeLV (Zoetis)	FeLVCHECK Ag ELISA (Agrolabo)	
		OD values	Results
45	NEG	0.051	NEG
46	NEG	0.041	NEG
47	POS	0.367	POS
48	NEG	0.045	NEG
49	POS	0.698	POS
50	POS	1.040	POS
51	POS	2.026	POS
52	POS	0.673	POS
53	NEG	0.039	NEG
54	NEG	0.040	NEG
55	POS	0.537	POS
56	POS	0.580	POS
57	NEG	0.073	NEG
58	NEG	0.039	NEG
59	NEG	0.037	NEG
60	NEG	0.068	NEG
61	NEG	0.044	NEG
62	POS	1.758	POS
63	NEG	0.138	NEG
64	NEG	0.040	NEG
65	POS	1.570	POS
66	POS	0.438	POS
67	POS	0.827	POS
68	POS	1.427	POS
69	POS	1.116	POS
70	POS	0.698	POS
71	NEG	0.040	NEG
72	POS	2.368	POS
73	POS	0.306	POS
74	POS	1.420	POS
75	POS	2.548	POS
76	NEG	0.053	NEG
**77***	**NEG**	**0.381**	**POS**
78	NEG	0.042	NEG
79	NEG	0.046	NEG
80	NEG	0.044	NEG
81	NEG	0.036	NEG
82	NEG	0.063	NEG
83	NEG	0.046	NEG
84	NEG	0.038	NEG
85	NEG	0.045	NEG
86	POS	1.604	POS
87	NEG	0.105	NEG
88	NEG	0.038	NEG
89	POS	0.371	POS
90	POS	0.539	POS
91	NEG	0.118	NEG

Continued in the next page

Supplementary Table S3: Continued.

Samples	ViraCHEK/FeLV (Zoetis)	FeLVCHECK Ag ELISA (Agrolabo)	
		OD values	Results
92	POS	2.684	POS
93	POS	2.076	POS
94	POS	0.835	POS
**95***	**NEG**	**0.987**	**POS**
96	POS	0.600	POS
97	POS	1.271	POS
**98***	**POS**	**0.201**	**DOUBTFUL**
99	POS	0.939	POS
100	NEG	0.035	NEG
101	NEG	0.037	NEG
102	NEG	0.037	NEG
103	POS	0.822	POS
104	NEG	0.068	NEG
105	NEG	0.047	NEG
106	NEG	0.037	NEG
107	NEG	0.034	NEG
108	NEG	0.033	NEG
109	POS	1.912	POS
110	POS	0.590	POS
111	NEG	0.036	NEG
112	NEG	0.038	NEG

Supplementary Table S4: Data for cut-off evaluation. Sp: Specificity; Se: Sensitivity; J: Jouden's index; PPV: Positive Predictive Value; NPV: Negative Predictive Value; LR+: positive likelihood ratio; LR-: negative likelihood ratio.

N. Cut-off	OD cut-off	Sp	1 - Sp	Se	Se-Sp	J	PPV	NPV	Accuracy	LR +	LR -	J max
1	0.030	0.000	1.000	1.000	1.000	0.000	0.393	-	0.393	1.000	-	0.971
2	0.040	0.471	0.529	1.000	0.529	0.471	0.532	1.000	0.670	1.892	0.000	
3	0.050	0.800	0.200	1.000	0.200	0.800	0.750	1.000	0.875	5.000	0.000	
4	0.060	0.829	0.171	1.000	0.171	0.829	0.778	1.000	0.893	5.833	0.000	
5	0.070	0.886	0.114	1.000	0.114	0.886	0.840	1.000	0.929	8.750	0.000	
6	0.080	0.900	0.100	1.000	0.100	0.900	0.857	1.000	0.938	10.000	0.000	
7	0.090	0.900	0.100	1.000	0.100	0.900	0.857	1.000	0.938	10.000	0.000	
8	0.100	0.900	0.100	1.000	0.100	0.900	0.857	1.000	0.938	10.000	0.000	
9	0.110	0.914	0.086	1.000	0.086	0.914	0.875	1.000	0.946	11.667	0.000	
10	0.120	0.943	0.057	1.000	0.057	0.943	0.913	1.000	0.964	17.500	0.000	
11	0.130	0.957	0.043	1.000	0.043	0.957	0.933	1.000	0.973	23.333	0.000	
12	0.140	0.971	0.029	1.000	0.029	0.971	0.955	1.000	0.982	35.000	0.000	
13	0.150	0.971	0.029	1.000	0.029	0.971	0.955	1.000	0.982	35.000	0.000	
14	0.160	0.971	0.029	1.000	0.029	0.971	0.955	1.000	0.982	35.000	0.000	
15	0.170	0.971	0.029	1.000	0.029	0.971	0.955	1.000	0.982	35.000	0.000	
16	0.180	0.971	0.029	1.000	0.029	0.971	0.955	1.000	0.982	35.000	0.000	
17	0.190	0.971	0.029	1.000	0.029	0.971	0.955	1.000	0.982	35.000	0.000	
18	0.200	0.971	0.029	1.000	0.029	0.971	0.955	1.000	0.982	35.000	0.000	
19	0.210	0.971	0.029	0.976	0.005	0.948	0.953	0.986	0.973	34.167	0.025	
20	0.220	0.971	0.029	0.976	0.005	0.948	0.953	0.986	0.973	34.167	0.025	
21	0.230	0.971	0.029	0.976	0.005	0.948	0.953	0.986	0.973	34.167	0.025	
22	0.240	0.971	0.029	0.976	0.005	0.948	0.953	0.986	0.973	34.167	0.025	
23	0.250	0.971	0.029	0.976	0.005	0.948	0.953	0.986	0.973	34.167	0.025	
24	0.260	0.971	0.029	0.976	0.005	0.948	0.953	0.986	0.973	34.167	0.025	
25	0.270	0.971	0.029	0.976	0.005	0.948	0.953	0.986	0.973	34.167	0.025	
26	0.280	0.971	0.029	0.976	0.005	0.948	0.953	0.986	0.973	34.167	0.025	
27	0.290	0.971	0.029	0.976	0.005	0.948	0.953	0.986	0.973	34.167	0.025	
28	0.300	0.971	0.029	0.976	0.005	0.948	0.953	0.986	0.973	34.167	0.025	
29	0.310	0.971	0.029	0.952	-0.019	0.924	0.952	0.971	0.964	33.333	0.049	
30	0.320	0.971	0.029	0.952	-0.019	0.924	0.952	0.971	0.964	33.333	0.049	
31	0.330	0.971	0.029	0.952	-0.019	0.924	0.952	0.971	0.964	33.333	0.049	
32	0.340	0.971	0.029	0.952	-0.019	0.924	0.952	0.971	0.964	33.333	0.049	
33	0.350	0.971	0.029	0.952	-0.019	0.924	0.952	0.971	0.964	33.333	0.049	
34	0.360	0.971	0.029	0.952	-0.019	0.924	0.952	0.971	0.964	33.333	0.049	
35	0.370	0.971	0.029	0.929	-0.043	0.900	0.951	0.958	0.955	32.500	0.074	
36	0.380	0.971	0.029	0.905	-0.067	0.876	0.950	0.944	0.946	31.667	0.098	
37	0.390	0.986	0.014	0.905	-0.081	0.890	0.974	0.945	0.955	63.333	0.097	
38	0.400	0.986	0.014	0.905	-0.081	0.890	0.974	0.945	0.955	63.333	0.097	
39	0.410	0.986	0.014	0.905	-0.081	0.890	0.974	0.945	0.955	63.333	0.097	
40	0.420	0.986	0.014	0.905	-0.081	0.890	0.974	0.945	0.955	63.333	0.097	
41	0.430	0.986	0.014	0.905	-0.081	0.890	0.974	0.945	0.955	63.333	0.097	
42	0.440	0.986	0.014	0.881	-0.105	0.867	0.974	0.932	0.946	61.667	0.121	
43	0.450	0.986	0.014	0.881	-0.105	0.867	0.974	0.932	0.946	61.667	0.121	
44	0.460	0.986	0.014	0.881	-0.105	0.867	0.974	0.932	0.946	61.667	0.121	
45	0.470	0.986	0.014	0.881	-0.105	0.867	0.974	0.932	0.946	61.667	0.121	
46	0.480	0.986	0.014	0.881	-0.105	0.867	0.974	0.932	0.946	61.667	0.121	
47	0.490	0.986	0.014	0.881	-0.105	0.867	0.974	0.932	0.946	61.667	0.121	
48	0.500	0.986	0.014	0.857	-0.129	0.843	0.973	0.920	0.938	60.000	0.145	
49	0.510	0.986	0.014	0.857	-0.129	0.843	0.973	0.920	0.938	60.000	0.145	
50	0.520	0.986	0.014	0.857	-0.129	0.843	0.973	0.920	0.938	60.000	0.145	
51	0.530	0.986	0.014	0.857	-0.129	0.843	0.973	0.920	0.938	60.000	0.145	
52	0.540	0.986	0.014	0.810	-0.176	0.795	0.971	0.896	0.920	56.667	0.193	
53	0.550	0.986	0.014	0.810	-0.176	0.795	0.971	0.896	0.920	56.667	0.193	
54	0.560	0.986	0.014	0.810	-0.176	0.795	0.971	0.896	0.920	56.667	0.193	

Continued in the next page

Supplementary Table S4: Continued.

N. Cut-off	OD cut-off	Sp	1 - Sp	Se	Se-Sp	J	PPV	NPV	Accuracy	LR +	LR -
55	0.570	0.986	0.014	0.810	-0.176	0.795	0.971	0.896	0.920	56.667	0.193
56	0.580	0.986	0.014	0.810	-0.176	0.795	0.971	0.896	0.920	56.667	0.193
57	0.590	0.986	0.014	0.786	-0.200	0.771	0.971	0.885	0.911	55.000	0.217
58	0.600	0.986	0.014	0.762	-0.224	0.748	0.970	0.873	0.902	53.333	0.242
59	0.610	0.986	0.014	0.738	-0.248	0.724	0.969	0.863	0.893	51.667	0.266
60	0.620	0.986	0.014	0.738	-0.248	0.724	0.969	0.863	0.893	51.667	0.266
61	0.630	0.986	0.014	0.738	-0.248	0.724	0.969	0.863	0.893	51.667	0.266
62	0.640	0.986	0.014	0.738	-0.248	0.724	0.969	0.863	0.893	51.667	0.266
63	0.650	0.986	0.014	0.738	-0.248	0.724	0.969	0.863	0.893	51.667	0.266
64	0.660	0.986	0.014	0.738	-0.248	0.724	0.969	0.863	0.893	51.667	0.266
65	0.670	0.986	0.014	0.738	-0.248	0.724	0.969	0.863	0.893	51.667	0.266
66	0.680	0.986	0.014	0.714	-0.271	0.700	0.968	0.852	0.884	50.000	0.290
67	0.690	0.986	0.014	0.714	-0.271	0.700	0.968	0.852	0.884	50.000	0.290
68	0.700	0.986	0.014	0.667	-0.319	0.652	0.966	0.831	0.866	46.667	0.338
69	0.710	0.986	0.014	0.667	-0.319	0.652	0.966	0.831	0.866	46.667	0.338
70	0.720	0.986	0.014	0.643	-0.343	0.629	0.964	0.821	0.857	45.000	0.362
71	0.730	0.986	0.014	0.643	-0.343	0.629	0.964	0.821	0.857	45.000	0.362
72	0.740	0.986	0.014	0.643	-0.343	0.629	0.964	0.821	0.857	45.000	0.362
73	0.750	0.986	0.014	0.643	-0.343	0.629	0.964	0.821	0.857	45.000	0.362
74	0.760	0.986	0.014	0.643	-0.343	0.629	0.964	0.821	0.857	45.000	0.362
75	0.770	0.986	0.014	0.643	-0.343	0.629	0.964	0.821	0.857	45.000	0.362
76	0.780	0.986	0.014	0.619	-0.367	0.605	0.963	0.812	0.848	43.333	0.386
77	0.790	0.986	0.014	0.619	-0.367	0.605	0.963	0.812	0.848	43.333	0.386
78	0.800	0.986	0.014	0.619	-0.367	0.605	0.963	0.812	0.848	43.333	0.386
79	0.810	0.986	0.014	0.619	-0.367	0.605	0.963	0.812	0.848	43.333	0.386
80	0.820	0.986	0.014	0.619	-0.367	0.605	0.963	0.812	0.848	43.333	0.386
81	0.830	0.986	0.014	0.548	-0.438	0.533	0.958	0.784	0.821	38.333	0.459
82	0.840	0.986	0.014	0.524	-0.462	0.510	0.957	0.775	0.813	36.667	0.483
83	0.850	0.986	0.014	0.524	-0.462	0.510	0.957	0.775	0.813	36.667	0.483
84	0.860	0.986	0.014	0.524	-0.462	0.510	0.957	0.775	0.813	36.667	0.483
85	0.870	0.986	0.014	0.524	-0.462	0.510	0.957	0.775	0.813	36.667	0.483
86	0.880	0.986	0.014	0.524	-0.462	0.510	0.957	0.775	0.813	36.667	0.483
87	0.890	0.986	0.014	0.524	-0.462	0.510	0.957	0.775	0.813	36.667	0.483
88	0.900	0.986	0.014	0.524	-0.462	0.510	0.957	0.775	0.813	36.667	0.483
89	0.910	0.986	0.014	0.524	-0.462	0.510	0.957	0.775	0.813	36.667	0.483
90	0.920	0.986	0.014	0.524	-0.462	0.510	0.957	0.775	0.813	36.667	0.483
91	0.930	0.986	0.014	0.524	-0.462	0.510	0.957	0.775	0.813	36.667	0.483
92	0.940	0.986	0.014	0.500	-0.486	0.486	0.955	0.767	0.804	35.000	0.507
93	0.950	0.986	0.014	0.500	-0.486	0.486	0.955	0.767	0.804	35.000	0.507
94	0.960	0.986	0.014	0.500	-0.486	0.486	0.955	0.767	0.804	35.000	0.507
95	0.970	0.986	0.014	0.500	-0.486	0.486	0.955	0.767	0.804	35.000	0.507
96	0.980	0.986	0.014	0.500	-0.486	0.486	0.955	0.767	0.804	35.000	0.507
97	0.990	1.000	0.000	0.500	-0.500	0.500	1.000	0.769	0.813	-	0.500
98	1.000	1.000	0.000	0.500	-0.500	0.500	1.000	0.769	0.813	-	0.500
99	1.010	1.000	0.000	0.500	-0.500	0.500	1.000	0.769	0.813	-	0.500
100	1.020	1.000	0.000	0.500	-0.500	0.500	1.000	0.769	0.813	-	0.500
101	1.030	1.000	0.000	0.500	-0.500	0.500	1.000	0.769	0.813	-	0.500
102	1.040	1.000	0.000	0.500	-0.500	0.500	1.000	0.769	0.813	-	0.500
103	1.050	1.000	0.000	0.476	-0.524	0.476	1.000	0.761	0.804	-	0.524
104	1.060	1.000	0.000	0.452	-0.548	0.452	1.000	0.753	0.795	-	0.548
105	1.070	1.000	0.000	0.452	-0.548	0.452	1.000	0.753	0.795	-	0.548

Supplementary Table S5: Reproducibility study. Each sample was tested in duplicate twice a day (4 tests/day) for 14 or 15 consecutive days (56 or 50 tests for each sample). PC: positive control (Protein G-HRP); NC: negative control (NC1: Pool of negative sera, 1:5000; NC2: PBS/BSA-based solution). Samples: positives (n.1, n.2), negatives (n.3, n.4); n.d.: not determined.

**Samples**	**Day 1**				**Day 2**				**Day 3**				**Day 4**			
	**OD 1**	**OD 2**	**OD 3**	**OD 4**	**OD 1**	**OD 2**	**OD 3**	**OD 4**	**OD 1**	**OD 2**	**OD 3**	**OD 4**	**OD 1**	**OD 2**	**OD 3**	**OD 4**
PC	3.900	3.965	4.000	4.000	3.900	3.966	3.868	3.946	3.858	3.973	3.970	4.000	3.740	3.816	3.877	3.907
NC1	0.051	0.058	0.052	0.055	0.061	0.062	0.058	0.066	0.056	0.061	0.057	0.061	0.055	0.060	0.054	0.056
NC2	0.050	0.050	0.050	0.051	0.048	0.050	0.050	0.049	0.052	0.051	0.052	0.051	0.051	0.050	0.050	0.052
Sample 1 (+)	1.463	1.407	1.284	1.291	1.324	1.344	1.311	1.330	1.397	1.334	1.397	1.508	1.437	1.327	1.388	1.400
Sample 2 (+)	0.814	0.871	0.880	0.846	0.826	0.815	0.706	0.749	0.789	0.768	0.749	0.765	0.710	0.670	0.733	0.790
Sample 3 (-)	0.056	0.055	0.055	0.056	0.051	0.052	0.062	0.064	0.060	0.058	0.059	0.054	0.054	0.050	0.055	0.059
Sample 4 (-)	0.040	0.037	0.039	0.041	0.043	0.045	0.039	0.038	0.041	0.042	0.042	0.040	0.044	0.041	0.041	0.046

**Samples**	**Day 5**				**Day 6**				**Day 7**				**Day 8**			
	**OD 1**	**OD 2**	**OD 3**	**OD 4**	**OD 1**	**OD 2**	**OD 3**	**OD 4**	**OD 1**	**OD 2**	**OD 3**	**OD 4**	**OD 1**	**OD 2**	**OD 3**	**OD 4**
PC	3.947	3.970	3.914	4.000	3.814	3.832	3.916	3.909	3.856	3.931	3.905	4.000	3.975	4.000	3.945	4.000
NC1	0.044	0.049	0.050	0.051	0.052	0.049	0.055	0.061	0.063	0.060	0.060	0.066	0.062	0.058	0.054	0.055
NC2	0.051	0.050	0.050	0.051	0.051	0.050	0.049	0.052	0.051	0.050	0.051	0.050	0.050	0.051	0.051	0.050
Sample 1 (+)	1.507	1.404	1.535	1.497	1.354	1.390	1.503	1.410	1.427	1.306	1.492	1.475	1.274	1.362	1.367	1.556
Sample 2 (+)	0.817	0.775	0.845	0.878	0.716	0.707	0.828	0.825	0.748	0.790	0.829	0.821	0.787	0.889	0.936	0.953
Sample 3 (-)	0.045	0.050	0.053	0.054	0.061	0.055	0.057	0.055	0.059	0.052	0.050	0.051	0.053	0.055	0.055	0.051
Sample 4 (-)	0.047	0.042	0.043	0.047	0.041	0.044	0.046	0.049	0.034	0.038	0.043	0.044	0.043	0.040	0.048	0.045

Continued in the next page

Supplementary Table S5: Continued.

Samples	Day 9				Day 10				Day 11				Day 12			
	**OD 1**	**OD 2**	**OD 3**	**OD 4**	**OD 1**	**OD 2**	**OD 3**	**OD 4**	**OD 1**	**OD 2**	**OD 3**	**OD 4**	**OD 1**	**OD 2**	**OD 3**	**OD 4**
PC	4.000	3.991	3.984	4.000	4.000	4.000	3.857	3.975	3.983	4.000	3.918	4.000	3.956	3.999	4.000	4.000
NC1	0.051	0.053	0.047	0.054	0.054	0.052	0.050	0.051	0.052	0.050	0.055	0.052	0.051	0.054	0.058	0.055
NC2	0.050	0.050	0.050	0.051	0.051	0.050	0.051	0.051	0.050	0.051	0.050	0.051	0.050	0.050	0.052	0.049
Sample 1 (+)	1.573	1.526	1.431	1.552	1.361	1.337	1.488	1.591	1.473	1.457	1.439	1.451	1.447	1.595	1.500	1.509
Sample 2 (+)	0.955	0.945	0.911	0.919	0.729	0.742	0.956	0.987	0.918	0.908	0.952	0.880	0.877	0.881	0.883	0.871
Sample 3 (-)	0.064	0.059	0.055	0.055	0.051	0.057	0.053	0.056	0.053	0.057	0.054	0.054	0.057	0.051	0.055	0.061
Sample 4 (-)	0.040	0.045	0.044	0.043	0.041	0.046	0.037	0.043	0.041	0.041	0.041	0.043	0.049	0.044	0.039	0.034

Samples	Day 13				Day 14				Day 15			
	**OD 1**	**OD 2**	**OD 3**	**OD 4**	**OD 1**	**OD 2**	**OD 3**	**OD 4**	**OD 1**	**OD 2**	**OD 3**	**OD 4**
PC	3.976	4.000	4.000	4.000	3.832	3.975	4.000	4.000	n.d	n.d	n.d	n.d
NC1	0.058	0.065	0.058	0.06	0.051	0.045	0.05	0.053	n.d	n.d	n.d	n.d
NC2	0.051	0.050	0.052	0.051	0.051	0.050	0.050	0.051	0.050	0.050	0.050	0.051
Sample 1 (+)	1.435	1.363	1.410	1.488	1.319	1.324	1.412	1.386	n.d	n.d	n.d	n.d
Sample 2 (+)	0.754	0.779	0.779	0.819	0.878	0.857	0.876	0.800	n.d	n.d	n.d	n.d
Sample 3 (-)	0.052	0.049	0.061	0.057	0.048	0.044	0.051	0.053	n.d	n.d	n.d	n.d
Sample 4 (-)	0.036	0.036	0.041	0.038	0.034	0.035	0.039	0.042	n.d	n.d	n.d	n.d

Supplementary Table S6: Reproducibility assay: intra- and inter-assay coefficient of variation. PC: positive control (protein G-HRP); NC: negative control (NC1: Pool of negative sera, 1:5000; NC2: PBS/BSA-based solution). In bold are highlighted the minimum and maximum %CVs. Samples: positives (n.1, n.2), negatives (n.3, n.4); N: number of assays; n.d.: not determined.

Day of analysis	N. assays	Intra-assay %CV							Inter-assay %CV						
		**PC**	**NC1**	**NC2**	**Sample 1**	**Sample 2**	**Sample 3**	**Sample 4**	**PC**	**NC1**	**NC2**	**Sample 1**	**Sample 2**	**Sample 3**	**Sample 4**
Day 1	1	1.169	9.082	0.000	2.759	4.784	1.274	5.510	0.584	6.524	0.700	1.572	3.785	1.274	4.523
	2	0.000	3.965	1.400	0.384	2.786	1.274	3.536							
Day 2	3	1.187	1.150	2.886	1.060	0.948	1.373	3.214	1.299	5.137	2.157	1.039	2.564	1.809	2.525
	4	1.412	9.124	1.428	1.017	4.179	2.245	1.837							
Day 3	5	2.077	6.044	1.373	3.262	1.907	2.397	1.704	1.305	5.419	1.373	4.333	1.701	4.327	2.577
	6	0.532	4.794	1.373	5.404	1.495	6.258	3.449							
Day 4	7	1.422	6.149	1.400	5.628	4.099	5.439	4.991	0.984	4.360	2.087	3.118	4.696	5.201	6.560
	8	0.545	2.571	2.773	0.609	5.293	4.962	8.128							
Day 5	9	0.411	7.603	1.400	5.004	3.731	7.443	7.945	0.974	4.502	1.400	3.388	3.220	4.382	7.115
	10	1.537	1.400	1.400	1.772	2.709	1.322	6.285							
Day 6	11	0.333	4.201	1.400	1.855	0.894	7.315	4.991	0.230	5.758	2.800	3.185	0.576	4.920	4.729
	12	0.127	7.315	4.201	4.515	0.257	2.525	4.466							
Day 7	13	1.362	3.449	1.400	6.261	3.862	8.918	7.857	1.531	5.092	1.400	3.536	2.274	5.159	4.741
	14	1.700	6.734	1.400	0.810	0.686	1.400	1.626							
Day 8	15	0.443	4.714	1.400	4.721	8.607	2.619	5.112	0.711	3.006	1.400	6.933	4.940	3.978	4.837
	16	0.979	1.297	1.400	9.144	1.273	5.337	4.562							
Day 9	17	0.159	2.720	0.000	2.145	0.744	5.749	8.319	0.221	6.261	0.700	3.941	0.681	2.874	4.972
	18	0.283	9.801	1.400	5.737	0.618	0.000	1.626							
Day 10	19	0.000	2.668	1.400	1.258	1.250	7.857	8.128	1.065	2.034	0.700	2.994	1.753	5.875	9.367
	20	2.131	1.400	0.000	4.731	2.256	3.892	10.607							
Day 11	21	0.301	2.773	1.400	0.772	0.774	5.143	0.000	0.883	3.369	1.400	0.680	3.166	2.571	1.684
	22	1.465	3.965	1.400	0.587	5.558	0.000	3.367							
Day 12	23	0.764	4.041	0.000	6.880	0.322	7.857	7.603	0.382	3.898	2.100	3.652	0.645	7.586	8.645
	24	0.000	3.755	4.201	0.423	0.968	7.315	9.686							
Day 13	25	0.426	8.048	1.400	3.639	2.306	4.201	0.000	0.213	5.223	1.387	3.723	2.923	4.497	2.685
	26	0.000	2.397	1.373	3.806	3.540	4.794	5.370							
Day 14	27	2.590	8.839	1.400	0.268	1.712	6.149	2.050	1.295	6.479	1.400	0.791	4.062	4.434	3.644
	28	0.000	4.119	1.400	1.314	6.413	2.720	5.238							
Day 15	29	n.d	n.d	0.000	n.d	n.d	n.d	n.d	n.d	n.d	0.700	n.d	n.d	n.d	n.d
	30	n.d	n.d	1.400	n.d	n.d	n.d	n.d							
**Minimum %CV**		**0.000**	**1.150**	**0.000**	**0.268**	**0.257**	**0.000**	**0.000**	**0.213**	**2.034**	**0.700**	**0.680**	**0.576**	**1.274**	**1.684**
**Maximum %CV**		**2.590**	**9.801**	**4.201**	**9.144**	**8.607**	**8.918**	**10.607**	**1.531**	**6.524**	**2.800**	**6.933**	**4.940**	**7.586**	**9.367**

Supplementary Table S7: Results of the comparative study. FeLVCHECK Ag ELISA (Agrolabo) was compared with INgezim FeLV DAS ELISA (Gold Standard Diagnostics) considering ViraCHEK/FeLV (Zoetis) as reference method. Discordant samples are marked with an asterisk and highlighted in bold. OD: optical density. GSD: Gold Standard Diagnostocs. POS: positive; NEG: negative.

Samples	ViraCHEK/FeLV (Zoetis)	FeLVCHECK Ag ELISA (Agrolabo)		INgezim FeLV DAS ELISA (GSD)	
		OD values	Result	OD values	Result
1	NEG	0.047	NEG	0.029	NEG
2	POS	2.612	POS	1.963	POS
3	POS	0.711	POS	0.300	POS
4	NEG	0.037	NEG	0.031	NEG
5	NEG	0.035	NEG	0.027	NEG
6	POS	1.466	POS	1.009	POS
7	NEG	0.114	NEG	0.060	NEG
8	POS	1.310	POS	0.853	POS
9	NEG	0.039	NEG	0.032	NEG
10	NEG	0.040	NEG	0.031	NEG
11	NEG	0.034	NEG	0.032	NEG
12	POS	1.126	POS	0.847	POS
13	NEG	0.036	NEG	0.031	NEG
14	NEG	0.043	NEG	0.031	NEG
15	NEG	0.040	NEG	0.03	NEG
16	NEG	0.046	NEG	0.033	NEG
17	NEG	0.047	NEG	0.028	NEG
18	NEG	0.035	NEG	0.029	NEG
19	NEG	0.038	NEG	0.027	NEG
20	POS	2.606	POS	2.106	POS
21	NEG	0.039	NEG	0.030	NEG
22	POS	1.363	POS	1.211	POS
23	NEG	0.046	NEG	0.036	NEG
24	NEG	0.046	NEG	0.037	NEG
25	NEG	0.037	NEG	0.027	NEG
26	NEG	0.036	NEG	0.029	NEG
27	NEG	0.036	NEG	0.029	NEG
28	NEG	0.039	NEG	0.033	NEG
29	POS	0.498	POS	0.425	POS
30	NEG	0.047	NEG	0.031	NEG
31	NEG	0.044	NEG	0.029	NEG
32	NEG	0.039	NEG	0.034	NEG
33	NEG	0.037	NEG	0.027	NEG
34	NEG	0.047	NEG	0.031	NEG
35	NEG	0.038	NEG	0.029	NEG
36	NEG	0.036	NEG	0.032	NEG
**37***	**NEG**	**0.129**	**NEG**	**0.282**	**POS**
38	NEG	0.037	NEG	0.029	NEG
39	NEG	0.064	NEG	0.041	NEG
40	NEG	0.038	NEG	0.029	NEG
**41***	**POS**	**0.779**	**POS**	**0.030**	**NEG**
42	POS	0.820	POS	0.380	POS
**43***	**NEG**	**0.036**	**NEG**	**0.352**	**POS**
44	POS	1.050	POS	0.587	POS

Continued in the next page

Supplementary Table S7: Continued.

Samples	ViraCHEK/FeLV (Zoetis)	FeLVCHECK Ag ELISA (Agrolabo)		INgezim FeLV DAS ELISA (GSD)	
		OD values	Result	OD values	Result
45	NEG	0.051	NEG	0.031	NEG
46	NEG	0.041	NEG	0.026	NEG
**47***	**POS**	**0.367**	**POS**	**0.188**	**NEG**
48	NEG	0.045	NEG	0.028	NEG
**49***	**POS**	**0.698**	**POS**	**0.255**	**NEG**
50	POS	1.040	POS	0.357	POS
51	POS	2.026	POS	0.811	POS
**52***	**POS**	**0.673**	**POS**	**0.223**	**NEG**
53	NEG	0.039	NEG	0.031	NEG
54	NEG	0.040	NEG	0.028	NEG
55	POS	0.537	POS	0.380	POS
**56***	**POS**	**0.580**	**POS**	**0.212**	**NEG**
57	NEG	0.073	NEG	0.040	NEG
58	NEG	0.039	NEG	0.027	NEG
59	NEG	0.037	NEG	0.030	NEG
60	NEG	0.068	NEG	0.034	NEG
61	NEG	0.044	NEG	0.032	NEG
62	POS	1.758	POS	0.693	POS
63	NEG	0.138	NEG	0.046	NEG
64	NEG	0.040	NEG	0.035	NEG
65	POS	1.570	POS	0.556	POS
**66***	**POS**	**0.438**	**POS**	**0.249**	**DOUBTFUL**
67	POS	0.827	POS	0.494	POS
68	POS	1.427	POS	0.720	POS
69	POS	1.116	POS	0.719	POS
70	POS	0.698	POS	0.305	POS
71	NEG	0.040	NEG	0.028	NEG
72	POS	2.368	POS	1.232	POS
73	POS	0.306	POS	0.228	POS
74	POS	1.420	POS	0.841	POS
75	POS	2.548	POS	1.243	POS
76	NEG	0.053	NEG	0.041	NEG
**77***	**NEG**	**0.381**	**POS**	**0.141**	**NEG**
78	NEG	0.042	NEG	0.029	NEG
79	NEG	0.046	NEG	0.029	NEG
80	NEG	0.044	NEG	0.033	NEG
81	NEG	0.036	NEG	0.039	NEG
82	NEG	0.063	NEG	0.048	NEG
83	NEG	0.046	NEG	0.035	NEG
84	NEG	0.038	NEG	0.041	NEG
85	NEG	0.045	NEG	0.047	NEG
86	POS	1.604	POS	0.867	POS
87	NEG	0.105	NEG	0.099	NEG
88	NEG	0.038	NEG	0.028	NEG
**89***	**POS**	**0.371**	**POS**	**0.127**	**NEG**
**90***	**POS**	**0.539**	**POS**	**0.172**	**NEG**
91	NEG	0.118	NEG	0.066	NEG

Continued in the next page

Supplementary Table S7: Continued.

Samples	ViraCHEK/FeLV (Zoetis)	FeLVCHECK Ag ELISA (Agrolabo)		INgezim FeLV DAS ELISA (GSD)	
		OD values	Result	OD values	Result
92	POS	2.684	POS	1.465	POS
93	POS	2.076	POS	1.027	POS
94	POS	0.835	POS	0.446	POS
95	NEG	0.987	POS	0.447	POS
96	POS	0.600	POS	0.290	POS
97	POS	1.271	POS	0.623	POS
98	POS	0.201	DOUBTFUL	0.108	NEG
99	POS	0.939	POS	0.336	POS
100	NEG	0.035	NEG	0.058	NEG
101	NEG	0.037	NEG	0.024	NEG
102	NEG	0.037	NEG	0.025	NEG
103	POS	0.822	POS	0.361	POS
104	NEG	0.068	NEG	0.043	NEG
105	NEG	0.047	NEG	0.049	NEG
106	NEG	0.037	NEG	0.036	NEG
107	NEG	0.034	NEG	0.031	NEG
108	NEG	0.033	NEG	0.084	NEG
109	POS	1.912	POS	0.816	POS
110	POS	0.590	POS	0.309	POS
111	NEG	0.036	NEG	0.044	NEG
112	NEG	0.038	NEG	0.044	NEG

Supplementary Table S8: Detailed data of accelerated stability study. At each time of analysis ($T_0 – T_6$) samples were tested in duplicate and the mean OD values and the percentage of remaining activities (% RA) were calculated. PC: positive control (protein G-HRP); NC: negative control (pool of negative sera, 1:5000). Samples: positives (1, 2), negatives (3, 4).

**Samples**	**T_0**				**T_1**				**T_2**				**T_3**			
	**OD 1**	**OD 2**	**Mean**	**% RA**	**OD 1**	**OD 2**	**Mean**	**% RA**	**OD 1**	**OD 2**	**Mean**	**% RA**	**OD 1**	**OD 2**	**Mean**	**% RA**
PC	3.255	3.265	3.260	100	3.250	3.257	3.254	99.80	3.216	3.212	3.214	98.59	3.185	3.176	3.181	97.56
NC	0.051	0.068	0.060	100	0.056	0.065	0.061	101.68	0.060	0.058	0.059	99.16	0.058	0.058	0.058	97.48
Sample 1 (+)	1.563	1.507	1.535	100	1.560	1.515	1.538	100.16	1.433	1.413	1.423	92.70	1.390	1.392	1.391	90.62
Sample 2 (+)	0.914	0.891	0.903	100	0.838	0.893	0.866	95.90	0.785	0.790	0.788	87.26	0.752	0.721	0.737	81.61
Sample 3 (-)	0.056	0.055	0.056	100	0.051	0.059	0.055	99.10	0.052	0.058	0.055	99.10	0.050	0.056	0.053	95.50
Sample 4 (-)	0.040	0.037	0.039	100	0.041	0.036	0.039	100.00	0.037	0.037	0.037	96.10	0.038	0.034	0.036	93.51

**Samples**	**T_4**				**T_5**				**T_6**			
	**OD 1**	**OD 2**	**Mean**	**% RA**	**OD 1**	**OD 2**	**Mean**	**% RA**	**OD 1**	**OD 2**	**Mean**	**% RA**
PC	3.054	3.094	3.074	94.29	2.952	2.958	2.955	90.64	2.834	2.842	2.838	87.06
NC	0.051	0.058	0.055	91.60	0.057	0.063	0.060	100.84	0.061	0.054	0.058	96.64
Sample 1 (+)	1.280	1.222	1.251	81.50	1.149	1.199	1.174	76.49	1.099	1.085	1.092	71.14
Sample 2 (+)	0.743	0.692	0.718	79.50	0.701	0.707	0.704	78.01	0.621	0.674	0.648	71.75
Sample 3 (-)	0.051	0.054	0.053	94.59	0.053	0.050	0.052	92.79	0.046	0.048	0.047	84.68
Sample 4 (-)	0.037	0.035	0.036	92.86	0.035	0.035	0.035	90.91	0.032	0.035	0.034	87.01

Supplementary Table S9: Detailed data of real-time stability study. At each time of analysis ($T_0 - T_{12}$) samples were tested in duplicate and the mean OD values and the percentage of remaining activities (% RA) were calculated. PC: positive control (protein G-HRP); NC: negative control (pool of negative sera, 1:5000). Samples: positives (1, 2), negatives (3, 4).

**Samples**	**T_0**				**T_1**				**T_2**				**T_3**			
	**OD 1**	**OD 2**	**Mean**	**% RA**	**OD 1**	**OD 2**	**Mean**	**% RA**	**OD 1**	**OD 2**	**Mean**	**% RA**	**OD 1**	**OD 2**	**Mean**	**% RA**
PC	3.255	3.265	3.260	100	3.255	3.261	3.258	99.94	3.200	3.266	3.233	99.17	3.257	3.297	3.277	100.52
NC	0.051	0.068	0.060	100	0.065	0.053	0.059	99.16	0.060	0.061	0.061	101.68	0.059	0.060	0.060	100.00
Sample 1 (+)	1.563	1.507	1.535	100	1.514	1.567	1.541	100.36	1.589	1.530	1.560	101.60	1.498	1.527	1.513	98.53
Sample 2 (+)	0.914	0.891	0.903	100	0.905	0.953	0.929	102.94	0.895	0.901	0.898	99.50	0.904	0.871	0.888	98.34
Sample 3 (-)	0.056	0.055	0.056	100	0.051	0.059	0.055	99.10	0.056	0.057	0.057	101.80	0.058	0.055	0.057	101.80
Sample 4 (-)	0.040	0.037	0.039	100	0.038	0.039	0.039	100.00	0.035	0.043	0.039	101.30	0.041	0.036	0.039	100.00

**Samples**	**T_4**				**T_5**				**T_6**				**T_7 (3 months)**			
	**OD 1**	**OD 2**	**Mean**	**% RA**	**OD 1**	**OD 2**	**Mean**	**% RA**	**OD 1**	**OD 2**	**Mean**	**% RA**	**OD 1**	**OD 2**	**Mean**	**% RA**
PC	3.249	3.289	3.269	100.28	3.254	3.241	3.248	99.62	3.267	3.253	3.260	100.00	3.167	3.184	3.176	97.41
NC	0.057	0.065	0.061	102.52	0.061	0.063	0.062	104.20	0.059	0.061	0.060	100.84	0.060	0.057	0.059	98.32
Sample 1 (+)	1.555	1.497	1.526	99.41	1.539	1.522	1.531	99.71	1.463	1.394	1.429	93.06	1.376	1.388	1.382	90.03
Sample 2 (+)	0.946	0.934	0.940	104.16	0.868	0.894	0.881	97.62	0.904	0.871	0.888	98.34	0.845	0.824	0.835	92.47
Sample 3 (-)	0.059	0.053	0.056	100.90	0.058	0.055	0.057	101.80	0.051	0.059	0.055	99.10	0.051	0.058	0.055	98.20
Sample 4 (-)	0.036	0.045	0.041	105.19	0.038	0.040	0.039	101.30	0.044	0.042	0.043	111.69	0.045	0.036	0.041	105.19

Continued on the next page

Supplementary Table S9: Continued.

Samples	T_8 (6 months)				T_9 (9 months)				T_{10} (12 months)				T_{11} (15 months)			
	**OD 1**	**OD 2**	**Mean**	**% RA**	**OD 1**	**OD 2**	**Mean**	**% RA**	**OD 1**	**OD 2**	**Mean**	**% RA**	**OD 1**	**OD 2**	**Mean**	**% RA**
PC	3.137	3.190	3.164	97.04	3.079	3.092	3.086	94.65	2.994	2.906	2.950	90.49	2.941	2.920	2.931	89.89
NC	0.062	0.063	0.063	105.04	0.052	0.067	0.060	100.00	0.054	0.066	0.060	100.84	0.054	0.055	0.055	91.60
Sample 1 (+)	1.429	1.429	1.429	93.09	1.390	1.322	1.356	88.34	1.372	1.327	1.350	87.92	1.281	1.270	1.276	83.09
Sample 2 (+)	0.828	0.871	0.850	94.13	0.823	0.850	0.837	92.69	0.806	0.847	0.827	91.58	0.740	0.784	0.762	84.43
Sample 3 (-)	0.057	0.050	0.054	96.40	0.051	0.053	0.052	93.69	0.051	0.055	0.053	95.50	0.050	0.051	0.051	90.99
Sample 4 (-)	0.035	0.042	0.039	100.00	0.037	0.040	0.039	100.00	0.044	0.035	0.040	102.60	0.032	0.034	0.033	85.71

Samples	T_{12} (18 months)			
	**OD 1**	**OD 2**	**Mean**	**% RA**
PC	2.819	2.820	2.820	86.49
NC	0.043	0.056	0.050	83.19
Sample 1 (+)	1.119	1.160	1.140	74.23
Sample 2 (+)	0.610	0.679	0.645	71.41
Sample 3 (-)	0.042	0.044	0.043	77.48
Sample 4 (-)	0.030	0.036	0.033	85.71

Supplementary Figure S1: Effect of different first incubation times. ELISA assays were performed by incubating the PC, samples (one positive and one negative) and conjugate antibody for 5, 10 and 15 minutes, then TMB for 5 minutes. Assays were carried out in duplicate. **A)** Visual results after addition of TMB; **B)** ELISA results expressed as mean OD values ± standard deviation (SD) after 5, 10, 15 minutes. PC: positive control (protein G-HRP 1:12,000); n: number of tests.

A)

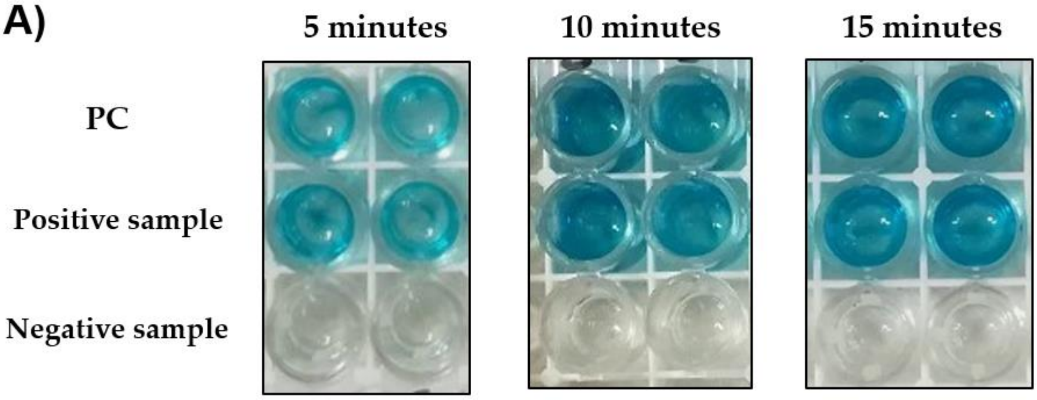

B)

Sample	5 minutes	10 minutes	15 minutes
	Mean OD ± SD	Mean OD ± SD	Mean OD ± SD
PC	1.251 ± 0.021 (n=2)	3.129 ± 0.021 (n=2)	3.210 ± 0.008 (n=2)
Positive sample	1.177 ± 0.026 (n=2)	2.611 ± 0.008 (n=2)	2.745 ± 0.005 (n=2)
Negative sample	0.065 ± 0.001 (n=2)	0.072 ± 0.002 (n=2)	0.075 ± 0.001 (n=2)
